# Roles of Cultivar, Light and Carbohydrates in Rooting of Cuttings of *Hydrangea macrophylla*

**DOI:** 10.3390/plants15060968

**Published:** 2026-03-20

**Authors:** Uwe Druege, Sindy Chamas

**Affiliations:** Erfurt Research Centre for Horticultural Crops, University of Applied Sciences Erfurt, 99090 Erfurt, Germany; sindy.chamas@fh-erfurt.de

**Keywords:** hortensia, cuttings, adventitious root, root development, light, dark, stress, carbohydrates, propagation

## Abstract

The roles of light and carbohydrates in adventitious root formation of *Hydrangea macrophylla* cuttings of the cultivars ‘Caipirinha’ and ‘Clarissa’ were investigated. Cuttings were planted immediately or dark-stored for seven days prior to cultivation under light. The leaf and rooting phenotype, relative chlorophyll content, carbohydrate levels in different cutting sections and rooting response to hexose were analyzed. Surprisingly, pronounced leaf yellowing and reddening and a strong hexose accumulation in the cutting leaves indicated that the hydrangea cuttings experienced light stress under a photosynthetic photon flux density (PPFD) of 100 µmol m^−2^ s^−1^. Reduction in PPFD to 50 µmol m^−2^ s^−1^ decreased these symptoms and increased chlorophyll content, but impaired rooting. The effects of dark storage depended on cultivar, PPFD, and hydration of cuttings. ‘Clarissa’ exhibited lower rooting success, particularly after dark storage and low light, and showed lower hexose-to-sucrose ratios and hexose concentrations in the stem base than ‘Caipirinha’. Rooting of ‘Clarissa’ could not be rescued by sugar supplementation, whereas application of 27 mM glucose plus 30 mM fructose for 24 h before planting enhanced rooting of ‘Caipirinha’. The lower hexose level in the stem base of ‘Clarissa’ does not appear to be the critical factor underlying its low rooting capacity.

## 1. Introduction

Hydrangea (*Hydrangea macrophylla* (Thunb.) Ser. is an economically important ornamental plant species because of its attractive foliage and its impressive floral display of many large, colorful inflorescences. Hydrangeas are mostly self-incompatible [1] and are propagated vegetatively via adventitious rooting of leafy shoot-tip cuttings. In this context, growers assessed the rooting capacity of the cultivar ‘Clarissa’ as low compared with other cultivars such as ‘Caipirinha’ (personal communication, Thomas Becker, company Kötterheinrich, Germany, 29 August 2019). In Germany, vegetative propagation of hydrangea, like that of many other perennial garden plants, is usually conducted at a single location, starting with stock-plant production and ending with the production of rooted cuttings. This stands in contrast to the propagation of many balcony plant species, such as petunia [2], whose cuttings are produced at distant stock plant facilities and experience at least short dark storage during their air transport to rooting stations in Europe. Depending on the species, those cuttings also experience extended dark storage when cuttings from sequential harvests are accumulated before shipping, which allows for maintaining smaller stock plant populations. Meanwhile, cultivation of stock plants in favorable climates, such as in tropical Africa, is also an interesting perspective for hydrangea due to the strongly increasing energy and labor costs in Europe.

Adventitious root (AR) formation in cuttings is a multiphasic, wound- and isolation-induced process that is commonly subdivided into the induction phase, during which specific AR source cells near the wound site are reprogrammed, the initiation phase, which starts with the appearance of the first new cell clusters and ends with the formation of dome-shaped AR primordia, and the expression phase, in which the complete root is formed, connected to the vascular system of the cutting, and emerges from the cutting [3,4]. AR formation is controlled by an array of endogenous factors, with prominent roles for plant hormones, particularly auxin, and for primary metabolites such as sugars, which provide energy and building blocks for the developing roots and, depending on their nature, can also function as signals [3,4,5]. Adventitious rooting of cuttings is further modulated by the plant genotype, epigenetic factors, and various environmental conditions at both the stock plant and cutting level, acting at multiple stages of the process.

Light and carbohydrates play critical roles in the AR formation of cuttings. The available surplus of carbohydrates in fully developed cutting leaves, serving as source organs, is important for supplying the stem base as a newly developing sink and depends on the initial carbohydrate levels at the time of planting and the current carbon gain during cutting cultivation [4]. For example, AR formation at the stem base of geranium was limited by carbohydrate shortages in leaves when dark-stored cuttings, depleted of their carbohydrate reserves, experienced low-light conditions during subsequent cultivation [6]. By contrast, enhancing net photosynthesis of the cuttings by increasing the light level during cultivation abolished the inhibitory effect of dark storage-induced leaf carbohydrate depletion at the time of planting, while the number of ARs was positively correlated with the mean sucrose level in the leaves and stem base during the first week of cultivation [7]. In petunia cuttings, the carbohydrate levels in basal leaves and stem base on day three after planting reflected the carbohydrate recovery status following the depletion caused by excision from stock plants and exacerbated by pre-planting dark storage [2,8]. Several other studies with diverse plant species have confirmed the important contribution of sufficiently high light intensity for AR formation and the positive relationships between light intensity or daily light integral, carbohydrate levels during rooting, and the final intensity of AR formation [9,10,11]. In addition to such carbohydrate source effects, carbohydrate sink activity also affects AR formation in cuttings. Thus, dark storage of petunia cuttings advanced the establishment of the new sink at the stem base when compared to the shoot apex through the local expression and activation of cell wall invertase, so that after planting, the assimilated carbon was preferentially directed toward the rooting zone [12]. However, none of these relationships has been investigated in hydrangea.

Our current research aims to characterize the rooting capacity of cuttings of the two hydrangea cultivars ‘Caipirinha’ and ‘Clarissa’ and to identify key environmental and endogenous control factors limiting adventitious rooting. As dark storage of hydrangea cuttings may gain importance in the future and the influence of light supply during rooting of hydrangea is under-investigated, in the present study, we analyzed the rooting response of the two cultivars to varying light conditions as modified by dark storage at different temperatures and by the PPFD during cutting cultivation. We hypothesized that the two cultivars differ in their rooting ability. We further hypothesized that adventitious rooting of hydrangea cuttings responds to light exposure and that modifications of carbohydrate source and/or sink are involved. In addition to the analysis of leaf vitality and rooting phenotype, we determined relative chlorophyll contents of leaves and analyzed concentrations and spatial distribution of carbohydrates within the cuttings. Based on these findings, we finally analyzed the rooting response of the two cultivars to increasing doses of specific sugars.

## 2. Results

### 2.1. Effects of Cultivar and Dark Storage on Leaf Performance, Adventitious Rooting and Carbohydrate Metabolism

In the first experiment, we characterized the rooting performance and carbohydrate metabolism of cuttings of the two cultivars by comparing immediately planted cuttings with those that had undergone a seven-day dark storage period at 20 °C or 4 °C before planting. This experimental setup was chosen to modify the balance between carbohydrate sources and sinks in the cuttings. Based on our experience with pelargonium cuttings [6], dark storage at 20 °C for one week was selected to impose pronounced dark stress on the cuttings and to permit substantial catabolic activity. The alternative exposure to 4 °C was chosen to reduce catabolic processes in darkness while still allowing metabolic activity and avoiding severe cold stress. Either immediately after harvest (unstored cuttings) or after removal from the storage room, cuttings were planted and cultivated for rooting in a climate chamber, where they were exposed to a PPFD of 100 µmol m^−2^ s^−1^ during a 16 h photoperiod. This relatively low PPFD was chosen because of the well-known semi-shade preference of *H. macrophylla.*

**Figure 1:**
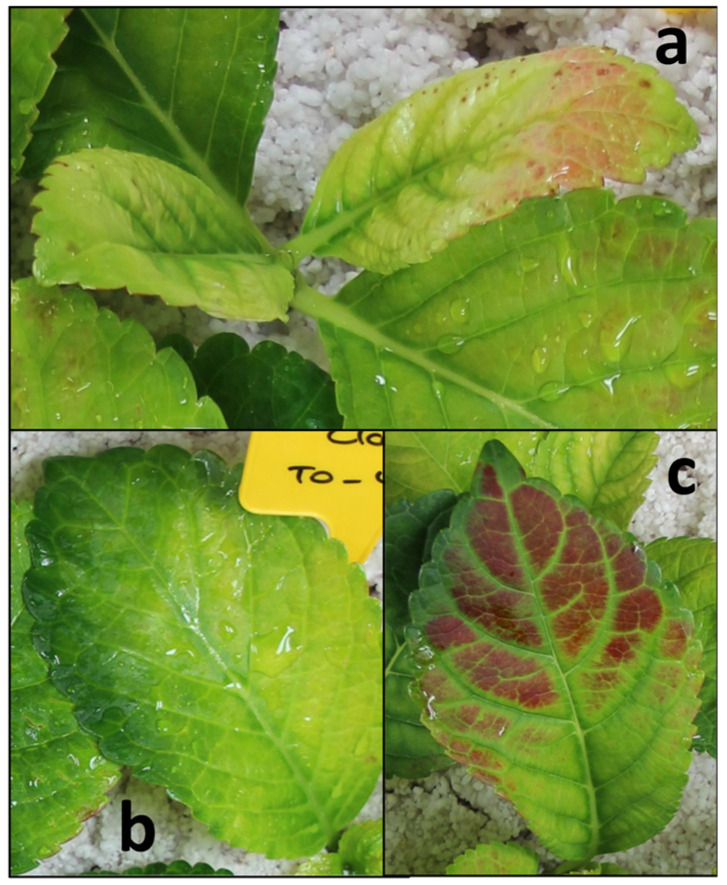
Leaf yellowing and reddening of exemplary young leaves (**a**) and basal leaves (**b**,**c**) of *H. macrophylla* ‘Caipirinha’ cuttings. Stages of the leaf in the upper right position of (**a**): Discoloration D3 (discolored leaf area > 2/3), yellowing D2 (yellow leaf area > 1/3 and ≤2/3), reddening D1 (red leaf area ≤ 1/3). Stages in (**b**): Discoloration and yellowing D3 (discolored and yellow leaf area > 2/3). Stages in (**c**): Discoloration D3 (discolored leaf area > 2/3), reddening D2 (red leaf area > 1/3 and ≤2/3), yellowing D1 (yellow leaf area > 1/3). The basal leaf in the upper left position in (**a**) and the apical leaf margins in (**b**) are normally green colored. Determined on day 30 post insertion into the substrate. Experiment 1.

Surprisingly, cuttings of both cultivars developed leaf discoloration when exposed to the low-light conditions of 100 µmol m^−2^ s^−1^. Discoloration included yellowing and reddening and affected the uppermost young leaves and basal leaves, as exemplarily shown in Figure 1 for the cultivar ‘Caipirinha’.

**Figure 2:**
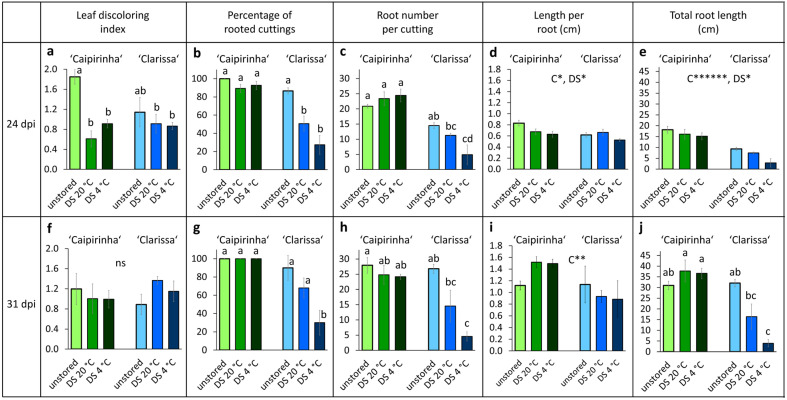
Leaf discoloring index (**a**,**f**), percentage of rooted cuttings (**b**,**g**), number (**c**,**h**), mean length (**d**,**i**) and total length (**e**,**j**) of roots formed at cuttings of *H. macrophylla* as affected by cultivar (C) and dark storage (DS) at two different temperatures, determined at 24 (**a**–**e**) and 31 (**f**–**j**) days after insertion (dpi) of cuttings into the rooting substrate. Mean values ± SE per combination of cultivar and dark storage. *, **, ******, indicate significant effects at *p* levels of 0.05, 0.01, 0.000001, respectively. In cases of significant interactions between cultivar and dark storage (see Supplementary Table S1), different letters a, b, c, d indicate significant differences between the specific combinations (*p* < 0.05, Tukey test, *n* = 5, each consisting of six cuttings). Experiment 1.

The visual ratings of leaf discoloration as the sum of yellowed and reddened leaf area and the intensity of AR formation analyzed at 24 and 31 days post insertion (dpi) of cuttings into the rooting substrate are illustrated in Figure 2, while the statistics of parameters are summarized in Supplementary Table S1. The data highlight a similar leaf discoloration for both cultivars, which was only transiently reduced by dark storage in ‘Caipirinha’ (Figure 2a,f; Supplementary Table S1), a higher rooting performance of ‘Caipirinha’ than of ‘Clarissa’, and a negative effect of dark storage on AR formation in both cultivars (Figure 2b–e,g–j; Supplementary Table S1). ‘Caipirinha’ outperformed ‘Clarissa’ in rooting percentage as well as in the mean and total numbers of ARs per planted cutting. Notably, the inhibitory effect of dark storage was more pronounced at 4 °C than at 20 °C. Based on significant cultivar × dark storage interactions, ‘Clarissa’s disadvantage in rooting percentage, root number and length became more pronounced after dark storage, particularly following low-temperature storage (Figure 2b,c,g,h,j).

**Figure 3:**
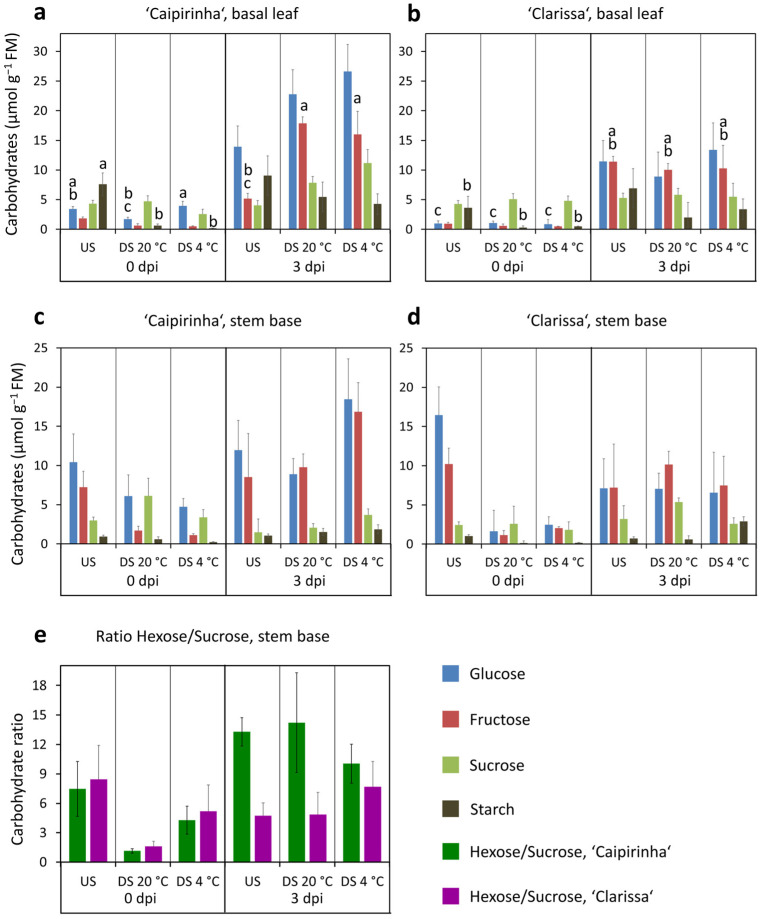
Carbohydrate levels in the basal leaf (**a**,**b**) and the stem base (1 cm, (**c**,**d**)) and the hexose/sucrose ratio in stem base (**e**) of *H. macrophylla* cuttings at zero days post insertion (dpi), the time of planting, and at 3 dpi as affected by cultivar and dark storage (DS) for seven days at two different temperatures. Mean values ± SE. In case of significant interactions between both factors according to Supplementary Table S2, different letters a, b, c indicate significant differences between the specific combinations for individual carbohydrates (*p* < 0.05, Tukey test, *n* = 6). US, unstored. Experiment 1.

In addition to assessing leaf color and rooting, we investigated early carbohydrate homeostasis in cuttings. Carbohydrate concentrations in basal leaves as the carbohydrate source organ and in the stem base as the establishing sink were analyzed at 0 dpi to assess the starting situation at planting and at 3 dpi to evaluate the early equilibrium after three days of exposure to the PPFD of 100 µmol m^−2^ s^−1^. Figure 3a–d provides an overview of glucose, fructose, sucrose, and starch concentrations across treatments and time, while Supplementary Table S2 summarizes the statistical effects of cultivar and dark storage. Carbohydrate analysis revealed a transient depletion by dark storage, a high accumulation of hexoses in leaves after planting, and lower early hexose availability in the stem base of ‘Clarissa’. Thus, dark storage reduced all carbohydrates in both cutting sections at the time of planting (0 dpi), leading to very low levels in leaves, except for leaf glucose and for sucrose (Figure 3a–d, Table 1, Supplementary Table S2). As a consequence, the ratio of hexose (sum of glucose and fructose) to sucrose in the stem base was lower after dark storage compared with the unstored cuttings (Table 1). After planting and exposure to the PPFD of 100 µmol m^−2^ s^−1^, the levels of glucose and fructose in the basal leaves showed a strong general increase between 0 dpi and 3 dpi (Figure 3a,b), whereas a much weaker or no increase was observed for sucrose and for the two hexoses in the stem base (Figure 3c,d). Nevertheless, cultivation under light counterbalanced the dark storage-induced carbohydrate depletion, so that at 3 dpi the previously dark-stored cuttings showed similar or even higher carbohydrate levels in both cutting sections than their immediately planted counterparts (Figure 3a–d, Supplementary Table S2).

The two cultivars revealed significant differences in their carbohydrate homeostasis. ‘Clarissa’, which showed lower rooting performance, was characterized by lower glucose levels in the basal leaf at 0 dpi and 3 dpi, and by higher sucrose, but lower glucose levels in the stem base at 3 dpi (Figure 3a–d, Supplementary Table S2). The hexose fraction in ‘Clarissa’ reached only 63% of ‘Caipirinha’ levels at 3 dpi, although this difference was non-significant with a *p* value of 0.07 (Table 1). Corresponding with the lower glucose and hexose availability, the hexose–sucrose balance in the stem base at 3 dpi was affected by cultivar, with ‘Clarissa’ showing a significantly lower hexose-to-sucrose ratio (Figure 3e, Table 1).

### 2.2. Effects of PPFD During Cutting Cultivation on Cultivar-Dependent and Dark Storage-Mediated Leaf Performance, Adventitious Rooting and Carbohydrate Metabolism

The combination of leaf discoloration with hexose overaccumulation after exposure to the PPFD of 100 µmol m^−2^ s^−1^ indicated light stress, causing energetic overload of the photosynthetic apparatus (see the discussion chapter). To test this hypothesis, we used the same basic experimental setup in experiment 2 but additionally compared a PPFD of 100 µmol m^−2^ s^−1^ with a 50% reduction to 50 µmol m^−2^ s^−1^ during cutting cultivation. We further differentiated between the two types of leaf discoloration (yellowing and reddening) and measured relative chlorophyll content in leaves using the SPAD method to assess the light response of the photosynthetic apparatus. Finally, we analyzed carbohydrates not only in the basal leaf and stem base but also in the shoot apex, which in cuttings constitutes an important utilization sink competing with the stem base [12].

**Figure 4:**
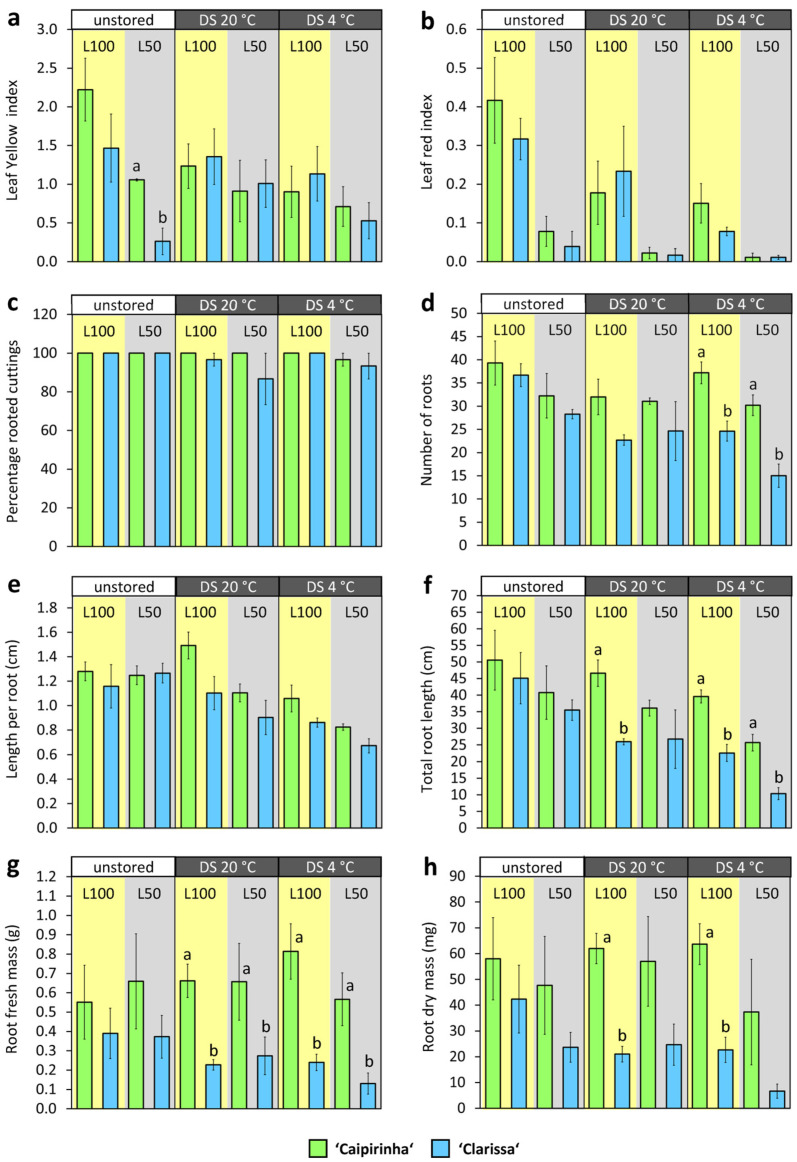
Leaf color (**a**,**b**) and rooting parameters (**c**–**h**) of cuttings of *H. macrophylla* as affected by cultivar, dark storage (DS) at two different temperatures and PPFD during cutting cultivation. Mean values ± SE per combination of cultivar, dark storage and PPFD (*n* = 3, each *n* consisting of 10 cuttings). Significant main effects of factors cultivar, dark storage, and PPFD are listed in Supplementary Table S3. Different letters a, b indicate significant differences between the two cultivars at the specific combination of dark storage and PPFD. L100, 100 µmol m^−2^ s^−1^; L50, 50 µmol m^−2^ s^−1^. Experiment 2.

The responses of leaf performance and rooting to cultivar, dark storage, and PPFD are illustrated in Figure 4, while the statistical effects are summarized in Supplementary Table S3. Confirming the hypothesis, a reduction in PFFD improved leaf performance but at the same time impaired rooting, while the data again highlighted the lower rooting performance of ‘Clarissa’ that was further dependent on the environmental factors, and a negative effect of dark storage on root formation (Figure 4).

Reducing PPFD to 50% decreased leaf yellowing and reddening (Figure 4a,b) to 54% and 13%, respectively (Supplementary Table S3). Dark storage also reduced leaf reddening, with stronger effects at low temperature, yielding the best leaf performance in cuttings stored at 4 °C and rooted at 50 µmol m^−2^ s^−1^ (Figure 4a,b). However, lowering PPFD significantly decreased the rooting performance (Figure 4c–h) so that low-PPFD cuttings produced only 84%, 86%, and 76% of the AR numbers, mean length, and total length recorded for high-PPFD cuttings (Supplementary Table S3).

**Figure 5:**
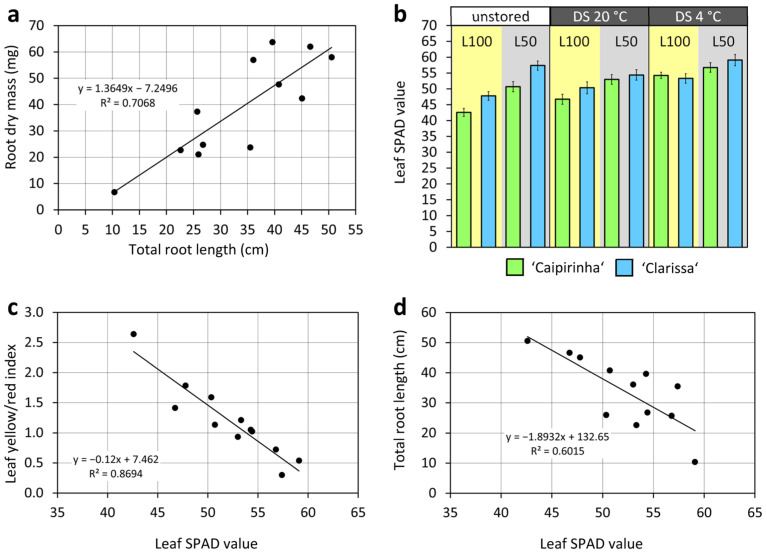
Relationship between total root length and root dry mass (**a**), relative chlorophyll contents of leaves (**b**), and relationships between leaf chlorophyll (**c**,**d**) and discoloration (**c**) and total root length (**d**) of cuttings of *H. macrophylla* under the influence of cultivar, dark storage (DS) at two different temperatures and PPFD during cutting cultivation. Mean values ± SE per combination of cultivar, dark storage and PPFD (*n* = 109–120). Regression slopes between the mean values per combination (*n* = 12) in (**b**–**d**). Statistics of SPAD levels are given in Supplementary Table S4. L100, 100 µmol m^−2^ s^−1^; L50, 50 µmol m^−2^ s^−1^. Experiment 2.

Confirming experiment 1, ‘Clarissa’ cuttings produced fewer and shorter ARs, as well as lower root fresh and dry mass, than ‘Caipirinha’ cuttings, while dark storage again reduced AR number and length (Figure 4, Supplementary Table S3). Furthermore, ‘Clarissa’s rooting disadvantage became more pronounced under additional stress factors. Although these interactions were not statistically significant, the lowest values for AR number, mean length, total length, fresh mass, and dry mass occurred in ‘Clarissa’ cuttings dark-stored at 4 °C and cultivated at 50 µmol m^−2^ s^−1^ (Figure 4d–h). Root dry mass was linearly correlated to total root length, which explained 71% of root dry mass variation across all combinations (Figure 5a).

Chlorophyll determination reinforced the positive effect of lower PPFD on leaf performance and further revealed the effects of cultivar and dark storage. Thus, dark storage slightly reduced relative chlorophyll content in leaves at planting (0 dpi) (Supplementary Table S4a). After 20 °C dark storage, ‘Clarissa’ exhibited higher leaf chlorophyll content than ‘Caipirinha’. Post-planting, relative chlorophyll content decreased until 3 dpi, while ‘Clarissa’ maintained higher levels than ‘Caipirinha’ (compare SPAD values in Supplementary Table S4a,b). Consistent with reduced leaf discoloration (Figure 4), both 4 °C dark storage and lower PPFD increased chlorophyll levels at 3 dpi (Figure 5b). Highest SPAD values were measured in ‘Clarissa’ cuttings that were either planted immediately or dark-stored at 4 °C and subsequently rooted under low PPFD (Figure 5b). The leaf discoloration index (integrating yellowed and reddened leaves) was strongly negatively correlated with relative chlorophyll levels (Figure 5c). Reflecting PPFD’s ambivalent effects on leaf discoloration and rooting (Figure 4), leaf chlorophyll content was negatively correlated with total root length (Figure 5d).

**Figure 6:**
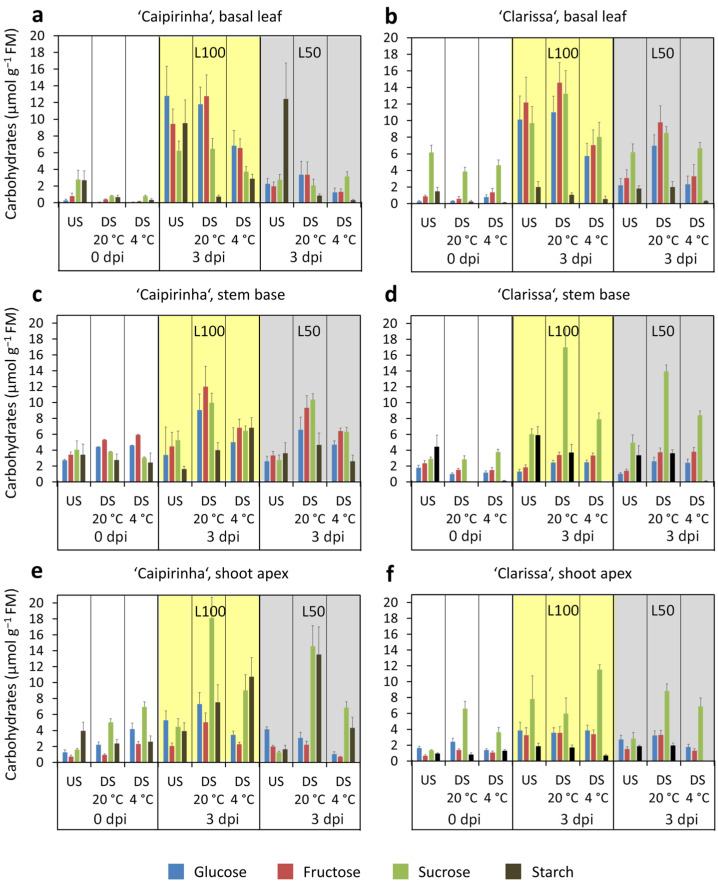
Carbohydrate levels in the basal leaf (**a**,**b**), the stem base (1 cm) (**c**,**d**) and the shoot apex (**e**,**f**) of *H. macrophylla* cuttings at zero days post insertion (dpi), the time of planting, and at 3 dpi as affected by cultivar and dark storage (DS) for seven days at two different temperatures, and PPFD during cutting cultivation. Mean values ± SE per combination (*n* = 12). Statistical results are listed in Supplementary Table S5. US, unstored cuttings; L100, 100 µmol m^−2^ s^−1^; L50, 50 µmol m^−2^ s^−1^. Experiment 2.

Figure 6 illustrates the carbohydrate levels in the basal leaf (a,b), the stem base (c,d), and the shoot apex (e,f) as affected by cultivar, dark storage and PPFD during cutting cultivation, while the statistics are summarized in Supplementary Table S5. Data confirmed most of the cultivar differences found in experiment 1, particularly the lower hexose level and hexose-to-sucrose ratio in the stem base of ‘Clarissa’, and further revealed a strong effect of PPFD on carbohydrate pools, which was much more pronounced in the basal leaves than in the other cutting sections.

**Figure 7:**
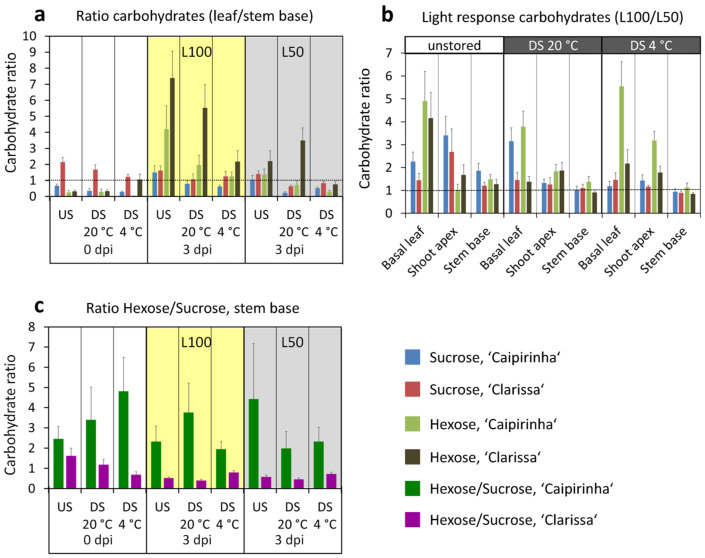
Ratios of carbohydrates between the basal leaf and stem base (**a**), the light response of carbohydrates in the different cutting sections as ratio of carbohydrate levels at L100/L50 (**b**), and the ratio between hexose and sucrose in the stem base (**c**) as affected by cultivar, dark storage (DS) for seven days at two different temperatures and the PPFD during cutting cultivation. Mean values ± SE (*n* = 12). Dashed lines indicate the level of ratio 1. Statistics are given in Supplementary Table S5. US, unstored; L100, 100 µmol m^−2^ s^−1^; L50, 50 µmol m^−2^ s^−1^. Experiment 2.

At planting (0 dpi), concentrations of most leaf sugars were higher in ‘Clarissa’ but glucose, fructose, hexose, and total sugar levels in the stem base were at a lower level than in ‘Caipirinha’ (Figure 6a–d). Independent of cultivar, dark storage at both temperatures reduced sucrose and total sugars in basal leaves, as well as starch levels in basal leaves and stem base (Figure 6a–d, Supplementary Table S5) By contrast, carbohydrate concentrations in the shoot apex either remained unaffected or even increased during dark storage (Figure 6e,f). Thus, shoot apices of ‘Caipirinha’ accumulated glucose, fructose, hexoses, sucrose, and total sugars during dark storage at 4 °C, reaching higher levels than ‘Clarissa’, whereas shoot apices of ‘Clarissa’ accumulated sucrose and total sugars during storage at 20 °C (Supplementary Table S5). At planting, sugar distribution between basal leaves and stem base differed markedly between cultivars: ‘Clarissa’ exhibited a much higher leaf-to-stem-base sucrose ratio than ‘Caipirinha’ (Figure 7a), and a significantly lower hexose-to-sucrose ratio in the stem base than ‘Caipirinha’, unaffected by storage (Figure 7c, Supplementary Table S5).

**Figure 8:**
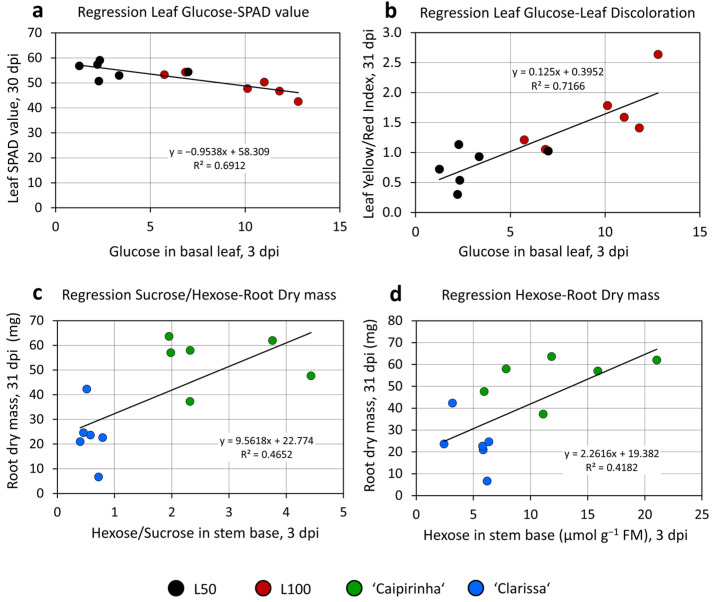
Regressions between the glucose level in the stem base at three days post insertion (dpi) and the relative chlorophyll content at 30 dpi, determined as SPAD value (**a**), the glucose level in the basal leaf at 3 dpi and leaf discoloration at 31 dpi (**b**), the hexose/sucrose ratio (**c**) and the hexose level (**d**) in the stem base at 3 dpi and the root dry mass produced until 31 dpi (**c**,**d**). Regression slopes between the mean values per combination (*n* = 12). L100, 100 µmol m^−2^ s^−1^; L50, 50 µmol m^−2^ s^−1^. FM, fresh mass. Experiment 2.

Independent of dark storage, reduction in PPFD after planting strongly reduced all sugar levels in basal leaves and shoot apex—but not in the stem base—at 3 dpi (Figure 6, Supplementary Table S5). To assess systemic PPFD effects on whole-cutting carbohydrate levels, we calculated light-response ratios for hexoses and sucrose in different cutting parts as sugar levels at high PPFD (L100) divided by those at low PPFD (L50). Figure 7b shows that sugars responded most strongly in basal leaves, less so in the shoot apex, and barely at all in the stem base. In this context, ‘Caipirinha’ exhibited stronger PPFD-induced increases in leaf sucrose, leaf hexoses, and even slight stem-base hexose elevation, whereas ‘Clarissa’ stem-base hexose remained unaffected by PPFD (Figure 7b, Supplementary Table S5). Notably, basal leaf sugar levels at 3 dpi negatively correlated with leaf chlorophyll content at 30 dpi and positively with leaf yellowing/reddening at 31 dpi (Supplementary Table S6), with glucose showing the strongest relationships. Thus, lowering glucose via reduced PPFD linearly increased chlorophyll content and decreased discoloration, explaining 69% and 72% of variations in chlorophyll level and discoloration, respectively (Figure 8a,b). Independent of dark storage and PPFD, ‘Clarissa’—as in experiment 1—showed at 3 dpi higher leaf fructose, sucrose, and total sugars, plus higher stem-base sucrose (Figure 6, Supplementary Table S5) but lower stem-base glucose, fructose, and hexoses than ‘Caipirinha’, with differences most pronounced after 20 °C dark storage (Figure 6c,d, Supplementary Table S5). As in experiment 1 (Figure 3e, Table 1), ‘Clarissa’ exhibited a significantly lower stem-base hexose-to-sucrose ratio at 3 dpi, unaffected by dark storage or PPFD (Figure 7c, Supplementary Table S5).

### 2.3. Relationships Between Sugar Metabolism and Adventitious Rooting of ‘Caipirinha’ and ‘Clarissa’

The correspondence of lower hexose levels, higher sucrose levels and lower hexose/sucrose ratio in the stem base at 3 dpi with the lower rooting performance of ‘Clarissa’ versus ‘Caipirinha’ indicated, that the lower rooting of ‘Clarissa’ was based on a lower availability of hexose in the stem base during the early phase of AR formation, possibly resulting from a lower conversion rate of arriving sucrose into glucose and fructose. This hypothesis was supported by positive relationships between the hexose/sucrose ratio or the hexose level in the stem base at 3 dpi as an independent variable and the dry mass of ARs formed until 31 dpi as a dependent variable (Figure 8c,d).

To test this hypothesis, we conducted two sugar response experiments. Prior to planting the cuttings in perlite, the stem bases of cuttings from both cultivars were immersed for 24 h in water solutions containing different concentrations of glucose plus fructose and compared to a sugar-free solution. The tested concentrations were selected based on the endogenous sugar levels measured in the stem base. Maximum fructose and glucose concentrations measured in the stem base of ‘Caipirinha’ were in a range around 10 µmol g^−1^ FM (Figure 6c). Assuming that one gram fresh mass contains ca. 900 µL (90%) of water, the molar concentration of each sugar would be 9 mM. Thus, for working at a physiological range but reaching also a strong surplus of sugars, both glucose plus fructose were applied in combination at concentrations of 9 + 10, 27 + 30, and 91 + 100 mM and the response of rooting under a PPFD of 50 µmol m^−2^ s^−1^ was analyzed (Based on a weighing error, the concentrations of both sugars were only approximately equimolar). To test also the particular sugar response of dark-stored cuttings, revealing lower endogenous sugar levels after storage (Figure 3 and Figure 6), both unstored cuttings and those dark-stored at 20 °C were compared. For each experiment, at the cultivar level, the effects of dark storage and sugar treatment were statistically analyzed.

**Figure 9:**
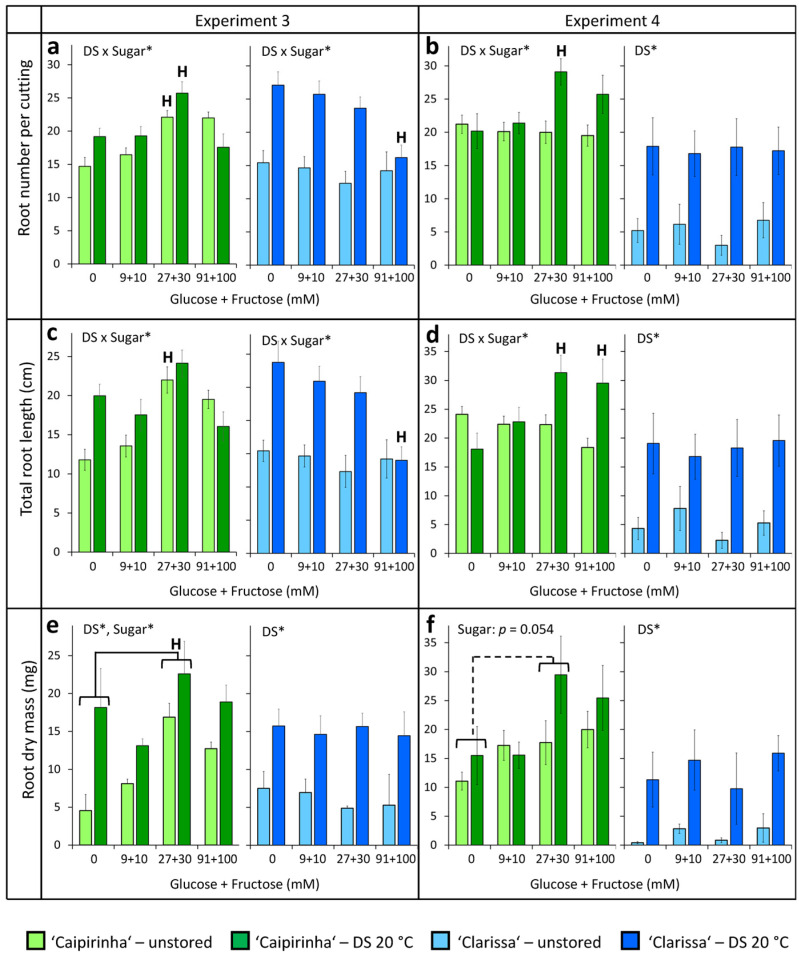
Response of rooting of unstored and dark-stored (DS, 7 days at 20 °C) cuttings of two *H. macrophylla* cultivars to increasing doses of glucose and fructose (indicated as mM per each sugar in water solutions) applied overnight for 24 h before planting. Number (**a**,**b**), length (**c**,**d**) and dry matter (**e**,**f**) of produced adventitious roots per planted cutting were determined at day 26 after planting. Results of two replicative experiments. Mean values ± SE. For each experiment and each cultivar, the effects of dark storage (DS) and sugar application (Sugar) were analyzed by two-factor ANOVA. Significant factors or interactions are indicated in each panel by asterisks (*p* < 0.05). H indicates a significant difference in the specific treatment to the sugar-free control (*p* < 0.05, Tukey test, *n* = 3 for root dry matter, *n* = 18 for the other parameters). Experiments 3 and 4.

Figure 9 illustrates the response of root number, total root length and root dry mass to the sugar applications, showing the results of both experiments side by side. Interestingly, no positive hexose response was observed for any rooting parameter of ‘Clarissa’. By contrast, in the first experiment, the highest supply of 91 + 100 mM hexose significantly decreased the number and total root length of those ‘Clarissa’ cuttings that had been previously exposed to dark storage (Figure 9a,c). In contrast to the absent or negative hexose response of ‘Clarissa’, ‘Caipirinha’ cuttings that already contained higher hexose levels revealed positive rooting responses to 27 + 30 mM or 91 + 100 mM hexose in both experiments. Thus, root number was enhanced by 27 + 30 mM hexose in both unstored and dark-stored cuttings in experiment 3 and in dark-stored cuttings in experiment 4 (Figure 9a,b). Similarly, the total root length of ‘Caipirinha’ was enhanced by 27 + 30 mM hexose in unstored cuttings in experiment 3 (Figure 9c), and by 27 + 30 mM and 91 + 100 mM hexoses in dark-stored cuttings in experiment 4 (Figure 9d).

In experiment 3, application of 27 + 30 mM hexose enhanced root dry mass independent of dark storage (Figure 9e). In experiment 4, a comparable effect was observed, but it was not statistically significant with a *p*-value of 0.054 (Figure 9f). Despite the positive correlations (Figure 8c,d), the hypothesis that the lower rooting capacity of ‘Clarissa’ versus ‘Caipirinha’ is based on the lower hexose concentrations in the stem during the early rooting phase was not supported by these findings. Obviously, hexose availability per se is not the primary limiting factor in ‘Clarissa’. By contrast, rooting of the better rooter ‘Caipirinha’, even though equipped with higher endogenous hexose levels, seems to have a higher demand for sugars, particularly after dark storage-induced sugar depletion, to realize maximum rooting.

## 3. Discussion

### 3.1. Hydrangea Cuttings Reveal a High Sensitivity of Leaves Against Light-Induced Energetic Overflow That Causes High Sugar Accumulation and Corresponding Senescence in Leaves

Considering the adequate fertilization of the stock plants and the type and distribution of observed leaf discolorations of the hydrangea cuttings when cultivated at a PPFD of 100 µmol m^−2^ s^−1^ (Figure 1), these did not indicate nutrient deficiency. Leaf yellowing in cuttings can be induced by dark-induced leaf senescence, as observed, for example, in dark-exposed pelargonium, where it has been linked to ethylene action and carbohydrate depletion [6,13]. However, in the present study, leaf discoloration was not exacerbated by dark storage (Figure 2) and was not associated with carbohydrate depletion (Figure 3a,b). The leaf yellowing and reddening (Figure 1 and Figure 2a,f) and strong leaf hexose accumulation (Figure 3a,b) observed at 100 µmol m^−2^ s^−1^, and the findings that both symptoms were reduced and chlorophyll contents were correspondingly increased by lowering the PPFD to 50% (Figure 4a,b, Figure 5b, Figure 6a,b and Figure 8a,b) strongly support the conclusion that even the relatively low PPFD of 100 µmol m^−2^ s^−1^ provide an energetic overflow of photosynthesis in hydrangea cuttings that causes chlorophyll breakdown and accumulation of red pigments as defense response to mitigate the light impact on the photosynthetic apparatus.

If excess light and the resulting excitation energy cannot be utilized downstream biochemically to assimilate CO_2_, photo-oxidative processes are triggered that produce diverse reactive oxygen species, which cause chloroplast and cell damage and leaf chlorosis [14,15,16]. Plants can adjust their leaf pigments to enhance their tolerance to excess light. This can involve the accumulation of anthocyanins and/or carotenoids, depending on the plant species. Reddening of leaves can result from the accumulation of anthocyanins, which play important roles in high-light adaptation, as they absorb visible and some UV light in the solar spectrum [17,18]. Alternatively, or in combination, carotenoids can accumulate in leaves under light stress. These pigments, which may appear red, orange, or yellow, can also mitigate light stress, since they absorb light in the range of approximately 400–550 nm [19,20]. In *H. macrophylla*, anthocyanins are the most important pigments coloring the sepals [21], while carotenoids appear to be less important [22]. However, no information has been published about their concentration and function in hydrangea leaves. Therefore, we currently cannot conclude which of these two pigment types may be responsible for leaf reddening in response to excess light.

It is remarkable that an energetic overload of photosynthesis is already initiated in *H. macrophylla* at such a low-light level of 100 µmol m^−2^ s^−1^, which permits only about 30% of maximum photosynthesis in petunia cuttings [23]. Recently, Li et al. [24] investigated the molecular response of two-year-old *H. macrophylla* ‘Endless Summer’ plants to variable light intensities in the field, comparing full shade, semi-shade, and unshaded conditions, which, however, involved much higher light levels than in the present study, with PPFDs of 351, 751, and 1503 µmol m^−2^ s^−1^, respectively. They found a complex light acclimation of leaves at the molecular level, involving genes regulating photosynthesis and carbohydrate metabolism, diverse transcription factors, and plant hormone homeostasis and signaling. Increasing light intensity from full shade to semi-shade reduced leaf size and enhanced the expression of several genes controlling the biosynthesis of jasmonic acid, of *AGPS1*, which controls starch biosynthesis, and of several genes of the *trehalose-6-phosphate synthase* (*TPS*) family [24]. TPS controls the biosynthesis of trehalose-6-phosphate, which responds to sucrose availability, induces starch biosynthesis, and inhibits starch breakdown [25,26]. This indicates that carbohydrate metabolism in the leaves of *H. macrophylla* plants is tightly regulated and highly sensitive to increasing light. Considering this generally high light sensitivity, the wounding of the cuttings in our study, which raises JA levels as shown for petunia [8], and their disconnection from the stock plants, have obviously further increased the light sensitivity of the hydrangea leaves.

Under excess light, carbohydrates tend to accumulate in leaves, although this light response depends on biochemical processes downstream of photosynthetic electron transport, including feedback regulation and carbohydrate export [27]. According to our findings, the accumulation of leaf sugars in response to excess light has been documented and related to leaf yellowing in various plant species. For example, detached leaves of barley (*Hordeum vulgare* L.) showed yellowing and sugar accumulation when exposed to 250–300 µE m^−2^ s^−1^ compared with 15–20 µE m^−2^ s^−1^ [28]. Similarly, leaves of wheat (*Triticum aestivum*) seedlings accumulated sugars and carotenoids and exhibited a decrease in chlorophyll levels when exposed to a PPFD of 800 µmol m^−2^ s^−1^ compared with 300 µmol m^−2^ s^−1^ [29,30]. Several studies indicate that high leaf sugar levels can trigger leaf senescence, while hexokinase (HXK), which regulates glucose entry into glycolysis and functions as a glucose sensor in sugar signaling processes [31], possibly acts as a mediator of the sugar signal. For instance, mature leaves of spinach (*Spinacia oleracea*) exhibited decreases in chlorophyll levels either when exposed to high light (300 µmol m^−2^ s^−1^ versus 10 µmol m^−2^ s^−1^), which caused sugar accumulation, including glucose, or when supplied with glucose via the transpiration stream [27]. Stem girdling in barley, which led to sugar accumulation in leaves, induced leaf yellowing and upregulation of senescence-associated genes [28]. Overexpression of *AtHXK1* in tomato accelerated leaf senescence [32], whereas leaf senescence in the *AtHXK1* mutant *gin2* was delayed compared with the wild type under glucose supply in the growth medium or under high light [31,33]. Considering these findings, the data of the present study support the hypothesis that the accumulation of sugars, particularly glucose, in hydrangea leaves under 100 µmol m^−2^ s^−1^ (Figure 3a,b and Figure 6a,b) not only reflects an energetic overload but also contributes to leaf senescence, resulting in a decrease in chlorophyll content and a corresponding increase in leaf discoloration (Figure 8a,b).

### 3.2. In Hydrangea Cuttings, the Capacity of Carbohydrate Transport Towards the Stem Base Is Low and Optimal Light Conditions Need to Find a Compromise Between Avoiding Leaf Damage and Favoring Adventitious Rooting

Our findings that the higher light level—despite the strong increase in leaf sugars—strongly enhanced the leaf/stem base ratio of sugars, particularly of hexoses (Figure 7a), but did not increase carbohydrates in the stem base (Figure 6c,d and Figure 7b), indicate that the carbon source capacity of the basal leaves exceeded the carbohydrate demand of the stem base that constitutes the newly developing sink [8]. Furthermore, the sink activity of the shoot apex appeared as higher than that of the stem base (Figure 7b). However, the finding that, despite the almost unaffected stem base sugars under 50 µmol m^−2^ s^−1^ compared to 100 µmol m^−2^ s^−1^, the lower light impaired rooting suggests that under the higher light, more carbohydrates were utilized for AR formation. Considering the light responses of leaf senescence and rooting, the optimum PPFD to avoid leaf senescence in hydrangea cuttings lies at a lower level than that required for maximal rooting. Further considering that a reduction in PPFD to 50 µmol m^−2^ s^−1^ did not completely prevent leaf discoloration (Figure 4a,b), the optimum PPFD for avoidance of leaf discoloration should be even lower than 50 µmol m^−2^ s^−1^.

Despite the relatively high R^2^ value of the negative SPAD–total root length relationship (Figure 5d), we consider this relationship not as causal but rather as being based on the two opposing effects of PPFD on leaf senescence and rooting. The high sensitivity of leaves to light stress implies only a limited potential for improving the carbohydrate supply of the rooting zone by higher light supply. However, finding ways to increase leaf tolerance to light stress would enable the use of higher PPFD without leaf damage and possibly enhance root formation in hydrangea cuttings. For example, given the important role of antioxidants in tolerance to excess light [18], their application may be one strategy to mitigate light stress in *H. macrophylla* cuttings.

### 3.3. Dark Storage Enhances Tolerance Against Light Stress of Hydrangea Cuttings and Modifies Rooting, While Temperature and Hydration Make a Difference

Our findings that dark storage of hydrangea cuttings reduced leaf discoloration and—after a transient depletion—enhanced chlorophyll levels during early rooting, particularly in combination with 4 °C (Figure 2a, Figure 4a,b and Figure 5b, Supplementary Table S4), indicate that tolerance against light stress of *H*. *macrophylla* can be enhanced by previous dark incubation. In another study, dark storage of rooted *H. macrophylla* cuttings (cultivar unspecified) at 15 °C caused transient depletion of leaf chlorophyll, which recovered upon re-exposure to light if the preceding dark period did not exceed nine days [34]. In that study, longer durations of dark storage induced progressive declines in chloroplast number and functional integrity, as evidenced by irregular morphology of granal and intergranal thylakoids and the presence of plastoglobuli. However, chloroplast morphology also recovered after 10 days under light when the dark period was ≤9 days [34]. Considering these findings, the chloroplasts in the present study may have undergone dark-induced remodeling that may have enhanced their flexibility for subsequent light acclimation compared with the non-prestressed chloroplasts of unstored cuttings, resulting in less discoloration upon re-exposure to light.

The found decrease in carbohydrate levels, particularly in basal leaves and to a lesser extent in the stem base during dark storage (Figure 3a–d and Figure 6a–d, Supplementary Tables S2 and S5) align with similar findings in cuttings of pelargonium, and petunia, where leaf carbohydrates were more sensitive to dark-induced depletion than those in the stem base [2,6]. This may reflect the inherently high dynamism and light responsiveness of leaf carbohydrates during the day/night cycle, with carbohydrate accumulation during the light period and mobilization during the dark phase [35]. It may also result from the transport of leaf-derived carbohydrates toward sinks in the cuttings, like the stem base, where a new sink can develop during dark storage through activation of invertases, as shown for petunia [12]. The stability of carbohydrate levels in the shoot apex during dark storage and the cultivar-dependent increase in specific sugars (Figure 6e,f, Supplementary Table S5) indicate that the apex exhibits a higher sink activity than the stem base during storage, which is probably higher in ‘Caipirinha’. The finding that the reduced carbohydrate levels in the basal leaf and stem base after dark storage were compensated for or even overcompensated during three days under light (Figure 3 and Figure 6), can be explained by current photosynthesis in the cuttings, which depends on current environmental factors such as PPFD, temperature, and CO_2_, on light acclimation of the photosynthetic apparatus prior to planting, and on the genotype [7,23].

The observation in experiments 1 and 2 that the dark storage-induced decrease in rooting was more pronounced when cuttings experienced 4 °C during storage compared with 20 °C, without consistent temperature effects on carbohydrates (Figure 2, Figure 3, Figure 4 and Figure 6, Supplementary Tables S1–S3 and S5), points to other inhibitory influences of the lower temperature. *H. macrophylla* plant development is temperature-sensitive, involving cold acclimation, dormancy or quiescence, and subsequent de-acclimation to survive frost periods in temperate zones [36,37]. The regulation of dormancy and quiescence involves plant hormones such as abscisic acid (ABA), whose production is stress-sensitive and which inhibits growth [38,39]. The current data may indicate that the one-week dark period at 4 °C already stimulated dormancy- or quiescence-related processes in the cuttings, reducing their physiological activity. Interestingly, ABA concentration in the xylem sap of *H. macrophylla* ‘Blaumeise’ plants increased over 8 days when cultivated under light at 4 °C compared with 20 °C [37]. Furthermore, ABA has been shown to inhibit adventitious rooting by suppressing cell cycle progression [3].

Interestingly, rooting of cuttings was not reduced but rather enhanced by dark storage at 20 °C, particularly in ‘Clarissa’, when the cuttings were hydrated before planting in experiments 3 and 4 (0 mM glucose + fructose in Figure 9). The data as a whole indicate that carbohydrate depletion during dark storage, discussed above, is not the dominant factor limiting rooting in hydrangea cuttings but rather water loss, which can be expected to occur when cuttings are kept in darkness after excision without hydration for several days [40]. The finding that ‘Clarissa’ was more severely affected by dark storage than ‘Caipirinha’ without hydration, but showed a stronger improvement in rooting with dark storage than ‘Caipirinha’ after hydration, indicates that ‘Clarissa’ is more sensitive to water deficit than ‘Caipirinha’ (compare dark storage responses of rooting parameters in Figure 2, Figure 4 and Figure 9). Most interestingly, a recent study with Arabidopsis and tomato explants provided evidence that higher local water availability at the site of potential root regeneration enhances root regeneration, while water availability shapes the spatial distribution of auxin response maxima [41]. The stimulation of rooting by dark storage at 20 °C, when water deficit was avoided (Figure 9), suggests that processes of AR induction and possibly root initiation occurred during dark storage. Such processes have been intensively investigated in petunia: when cuttings of *Petunia hybrida* ‘Mitchell’ were dark-stored for one week at 10 °C and compared with immediately planted cuttings, cell wall invertase activity, which determines sink strength, was activated, and initial cell clusters were formed by the end of dark storage [2,12].

### 3.4. ‘Clarissa’ Shows a Lower Rooting Capacity, a Higher Sensitivity to Post-Harvest Stress and a Lower Capacity to Utilize Sucrose in the Stem Base, but Apparently Other Factors Limit the Rooting

Across all experiments, the rooting data reflect a lower rooting capacity of ‘Clarissa’ compared with ‘Caipirinha’ (Figure 2 and Figure 4, Supplementary Tables S1 and S3). However, the overall data variation reveals that this rooting disadvantage relative to ‘Caipirinha’ increases as additional stressors, such as dark storage without hydration and low PPFD, further limit rooting (Figure 2 and Figure 4). The exhibited lower hexose/sucrose ratios and lower hexose (particularly glucose) concentrations, as well as the higher sucrose levels, in the stem base of ‘Clarissa’ compared with ‘Caipirinha’ (Table 1, Figure 3c–e, Figure 6c,d and Figure 7c; Supplementary Table S5) clearly demonstrate that the capacity to hydrolyze incoming sucrose to glucose and fructose in the newly developing stem base sink [8] is lower than in ‘Caipirinha’. This suggests lower activities of enzymes, which convert sucrose to glucose plus fructose. In this context, invertases have been shown to play particular roles in sink strength [42,43] and in sink establishment during AR formation in petunia [8,12].

While the apparently lower sucrose utilization in the stem base of ‘Clarissa’ can be expected to cause upstream a lower influx of sucrose compared with ‘Caipirinha’ based on feedforward mechanisms [43], the resulting lower hexose levels in the stem base could limit AR development, which may result from deficiencies in energy and/or carbon skeletons as well as from sugar signaling effects. Sugar signaling effects during AR formation may involve HXK as a glucose sensor, as recently discussed [4]. The low R^2^ values of the relationships between the sucrose–hexose ratio or the hexose levels, and root dry mass (Figure 8c,d) already indicated that other factors are also involved in the different rooting capacity of the two cultivars. The finding that root formation in ‘Clarissa’, unlike in ‘Caipirinha’, could not be stimulated but was in some cases even inhibited by external hexose supply (Figure 9) reveals that lower endogenous hexose levels per se are not the dominant factor limiting rooting capacity in this cultivar. Rather, other factors, possibly hormonal, appear more critical. Among these, auxin, an important inducer, and/or ABA, a potential inhibitor, of adventitious rooting may play key roles [3,4], as both are particularly sensitive to water availability [41,44], which appears to play an important role in hydrangea as discussed above.

Interestingly, root formation in ‘Caipirinha’, the stronger rooting cultivar, was stimulated by 27 + 30 mM glucose + fructose irrespective of its higher endogenous hexose levels, whereas this effect was most consistent in combination with dark storage at 20 °C (Figure 9). This suggests that suboptimal endogenous hexose supply in ‘Caipirinha’ becomes the dominant limiting factor for adventitious rooting under additional low-light stress.

## 4. Materials and Methods

### 4.1. Plant Material

Stock plants of *Hydrangea macrophylla* (Thunb.) Ser. ‘Caipirinha’ and ‘Clarissa’ were grown in a greenhouse at the Erfurt Research Centre of Horticultural Crops. In total, four experiments were conducted. For experiments 1 and 2, stock plants were established in spring 2019 from young plants obtained from the company Kötterheinrich-Hortensienkulturen, Lengerich, Germany. For experiments 3 and 4, young plants were obtained in spring 2020 from a commercial supplier of in vitro-propagated young plants. The young plants were stepwise potted into 12 cm and then 17 cm pots using a peat-based substrate enriched with iron (CL ED73 + Eisen + pH; Patzer, Sinntal, Germany). During the growth period between week 10 and week 46, greenhouse day/night temperatures were maintained at 20/22 °C (heating) and 22/20 °C (ventilation), respectively. A 16 h photoperiod was provided by high-pressure sodium lamps, with shading activated when outdoor radiation exceeded 30 klx. From late autumn (week 47), stock plants were cut back and kept frost-free at low temperatures (heating/venting at 2 °C/4 °C) under natural photoperiods to induce a quiescent period. In spring (week 10), after fulfilling the 1000 h × 4 °C cold requirement, plants were returned to the above temperature and light conditions to promote growth and development of cuttings for the next growth season. During the growth season, depending on substrate analysis results, liquid fertilization (0.1–0.3% Hakaphos Soft Spezial 16-8-22(+3); Compo, Münster, Germany) was applied up to three times weekly. Target N_min_ content in the substrate was 100–150 mg L^−1^.

### 4.2. Experimental Design

In experiment 1, the two factors cultivar (two levels: ‘Caipirinha’, ‘Clarissa’) and dark storage (three levels: unstored, dark-stored at 20 °C, dark-stored at 4 °C) were combined, and leaf performance, AR formation, and carbohydrate dynamics in cuttings were analyzed. Considering the semi-shade preference of *H. macrophylla*, a relatively low PPFD of 100 µmol m^−2^ s^−1^ was used during cutting cultivation. In experiment 1, leaf performance and sugar accumulation indicated light stress already at 100 µmol m^−2^ s^−1^. Therefore, experiment 2 compared two PPFD levels, 100 µmol m^−2^ s^−1^ versus a 50% reduction to 50 µmol m^−2^ s^−1^, as a third factor in combination with the two others. In addition to the parameters of experiment 1, the relative chlorophyll content of leaves was also analyzed. Because experiments 1 and 2 pointed to an important role of hexoses in AR formation of *H. macrophylla*, in experiments 3 and 4, the influence of hexose application (four levels) on AR formation was investigated at the cultivar level in combination with dark storage (two levels: unstored versus dark stored at 20 °C).

### 4.3. Harvest and Treatment of Cuttings

Shoot tip cuttings (softwood cuttings), consisting of a stem of ca. 3 cm length with two nodes bearing one pair of fully developed leaves, two younger leaves, and the shoot apex, were excised at regular intervals from week 14 onwards until the start of the quiescent period, always leaving two leaves or nodes on the stock plant to promote axillary shoot growth. For experiments 1, 2, 3 and 4, in total 432, 540, 288 and 288 cuttings were harvested on 11 August and 10 November 2020, 10 November 2021 and 17 May 2022.

Standard rooting: Cuttings were immediately planted in trays containing Perligran A perlite (Knauf Perlite GmbH, Dortmund, Germany). The trays for experiments 1, 2, 3 and 4 were 35.3 × 55.3 × 5.7 cm^3^, 28.3 × 46.5 × 5.7 cm^3^, and 20.5 × 33.0 × 5.8 cm^3^ in size and contained 24, 20, and 12 cuttings, respectively. In each tray, the same number of cuttings from both cultivars were planted. Cuttings were cultivated in a growth chamber under the following conditions: temperature, 22/20 °C (day/night); humidity outside covered trays, 85/60% (day/night). Depending on the experiment, photosynthetic photon flux density (PPFD) was 100 µmol m^−2^ s^−1^ (L100) or 50 µmol m^−2^ s^−1^ (L50) at the plant level during a 16 h photoperiod (5:00 a.m.–9:00 p.m.) provided by white, fluorescent tubes. Variation in PPFD along the benches was ±10% of the mean value, based on five measurements, including the minimum and maximum values under the edges and the center of the illumination units. To cover possible slight variations in light, temperature, and humidity along the benches resulting from illumination and air flow, the trays were systematically oriented along the benches in such a way that positions below the center and edges of the two illumination units per bench were equally distributed among the cultivars and storage treatments. Trays were covered with transparent plastic hoods, including venting holes to create a microclimate with increased humidity. During the rooting period, cuttings were manually watered and did not receive nutrients or phytohormones.

Dark storage: As an alternative to immediate planting, cuttings were enclosed in polyethylene bags, packed in cardboard boxes, and stored for seven days in a dark cabinet at 20 °C or 4 °C before being planted and cultivated as described above.

Hexose applications: To analyze the rooting response to glucose and fructose in two experiments, cuttings were placed with their stem bases in aqueous solutions containing different near-equimolar concentrations of glucose (Duchefa Biochemie, Haarlem, The Netherlands) and fructose (Carl Roth GmbH + Co. KG, Karlsruhe, Germany) and incubated overnight (24 h) under the described growth chamber conditions. Thereafter, cuttings were planted and cultivated as described for standard rooting. Due to a calculation error, the glucose:fructose molar ratio was 0.91. Thus, glucose and fructose concentrations of 9 + 10, 27 + 30, and 91 + 100 mM were compared with a sugar-free control (0 mM). All solutions contained chloramphenicol (98 mg L^−1^, Duchefa Biochemie) and nystatin (35 mg L^−1^, Sigma Aldrich, Merck KGaA, Darmstadt, Germany) to prevent microbial growth.

### 4.4. Assessment of Leaf Vitality and Rooting Performance

At the end of the rooting periods, leaf discoloration and rooting were assessed by trained persons. Based on the high number of cuttings that had to be scored (up to 240 per day in experiment 2), this work was shared among several people. To avoid bias between the cultivars and treatments, each person assessed a specific replication tray of all treatments.

The extent of leaf discoloration (yellowing and reddening) was determined in experiment 1 at 24 and 31 dpi and in experiment 2 at 31 dpi. In experiment 1, leaf yellowing and reddening were not distinguished from each other. In experiment 2, leaf reddening and yellowing were assessed separately. The number of leaves showing yellowing or reddening was assessed by classifying leaves into three discoloration categories: D1, discolored/yellow/red leaf area ≤ 1/3; D2, discolored/yellow/red leaf area > 1/3 and ≤2/3; D3, discolored/yellow/red leaf area > 2/3). Examples of these ratings are illustrated in Figure 1. Using midpoints of the three classes, the leaf discoloration index (DI) per cutting was calculated as: DI = (D1 × 0.167 + D2 × 0.5 + D3 × 0.833).

At 24 and 31 dpi, 31 dpi and 26 dpi, the intensity of AR formation was assessed in experiments 1, 2, 3 and 4, respectively. The percentage of rooted cuttings was calculated by dividing the number of rooted cuttings per cultivar and tray by the number of planted cuttings. Roots of each cutting were counted and assigned to different root length classes (intervals of 1 cm). To calculate root length values, the midvalues of the respective classes were used. For example, roots measuring 2–3 cm were assigned a value of 2.5 cm. Average root number and total root length per planted cutting were calculated by dividing the sum of roots and the sum of length midvalues, respectively, by the number of planted cuttings. Mean root length was calculated by dividing the total root length by the root number. In Experiments 2–4, the fresh and dry mass of roots were determined. Before the gravimetric determination of root fresh mass, roots were removed from the cuttings and carefully dried by gentle pressing between paper towels until the paper no longer absorbed any water. Subsequently, dry mass was determined gravimetrically after drying for 24 h at 60 °C. In Experiments 1 and 2, and for the calculation of root dry matter in Experiments 3 and 4, each tray represented a biological replicate with *n* = 5, *n* = 3, and *n* = 3, while each *n* consisted of 6, 10, and 6 cuttings, respectively. For the other parameters in Experiments 3 and 4, individual cuttings were considered as biological replicates (*n* = 18).

### 4.5. Determination of Relative Chlorophyll Content

Based on established linear relationships between SPAD values and chemically determined chlorophyll concentrations in leaves of Arabidopsis and 30 ornamental plant species [45,46], relative chlorophyll content in leaves was determined in experiment 2 at 0 dpi and 3 dpi non-invasively using the SPAD-502 chlorophyll meter (Konica Minolta, Osaka, Japan). The device measures leaf transmittance at red (650 nm; measuring wavelength) and infrared (940 nm; reference wavelength) wavelengths and provides an output proportional to leaf chlorophyll concentration. Measurements were taken from all leaves per cutting, targeting the most chlorotic leaf areas visually. At 0 dpi and 30 dpi, in total 238–240 and 109–120 measurements (*n*) were conducted on 60 and 30 cuttings per combination of cultivar, storage and light treatment.

### 4.6. Analyses of Carbohydrates

Samples for carbohydrate analysis were collected at 0 dpi and at 3 dpi, in case of light-exposed cuttings (0 dpi: unstored, 3 dpi: all), at 5 h after the onset of the photoperiod. Depending on the experiment, 1 cm long stem base segments, three leaf disks from the central region between the middle rib and margin of basal leaves, and the shoot apex, including the smallest adjacent leaves, were excised with a scalpel, weighed, immediately shock-frozen in liquid nitrogen, and stored at −80 °C until analysis. Carbohydrate measurements in two sections (stem base, basal leaves) from 6 cuttings as biological replicates (*n* = 6) in experiment 1 and in three sections (stem base, basal leaves, shoot apex) from 10 cuttings as biological replicates (*n* = 10) in experiment 2 were done. The different time points, cultivars and treatments resulted in 144 and 540 samples from 72 and 180 cuttings in experiments 1 and 2, respectively.

Carbohydrates were analyzed enzymatically modified after Klopotek et al. [2]. Used enzymes and sugar standards were from Sigma Aldrich Merck KGaA and other chemicals from Carl Roth GmbH + Co. KG. Frozen samples were ground in a vibration mill (Retsch, Haan, Germany), extracted in 80% ethanol + 20% imidazole buffer (10 mM, pH 6.9) for 1 h at 80 °C, purified using 100 mg activated carbon per sample, and centrifuged at 15,000 rpm at 4 °C. Supernatants were pipetted into microplates (Anicrin, Scorzè, Italy) and dried overnight at 37 °C. Sugars were quantified photometrically by measuring NADH + H^+^ at 340 nm in a FLUOstar Omega microplate reader (BMG Labtech, Ortenberg, Germany). NADH + H^+^ production is proportional to sugar concentration through sequential conversion of glucose, fructose, and sucrose to glucose-6-phosphate following the addition of hexokinase and glucose-6-phosphatase, glucose-6-phosphate isomerase, and invertase, respectively, and final conversion of glucose-6-phosphate to 6-phosphogluconate, which results in conversion of NADH into NADH + H^+^. For starch analysis, centrifugation pellets were treated with KOH, and the resulting starch fragments were hydrolyzed to glucose using amyloglucosidase. Glucose concentrations were then measured as described above.

### 4.7. Statistics

Mean values and standard errors per treatment were calculated using Microsoft Excel Version 2108 (Microsoft, Redmond, WA, USA). Effects of cultivar, dark storage, light intensity, and sugar application were analyzed by two- or three-way ANOVA using TIBCO Statistica 13.3 (TIBCO Software Inc., Palo Alto, CA, USA). Significance was calculated at *p* levels of 0.05, 0.01, 0.001, 0.0001, 0.00001 and 0.000001. Significant differences between means were identified using the Tukey HSD test (*p* < 0.05). The number of replications is provided with the data. Slopes and coefficients of determination (R^2^) of linear regressions were calculated using the trendline function of Microsoft Excel.

## 5. Conclusions

Cuttings of *H. macrophylla* are highly sensitive to light stress, responding by leaf hexose overaccumulation and discoloration even at a PPFD of 100 µmol m^−2^ s^−1^. The PPFD during cultivation of *Hydrangea* cuttings should be kept below 50 µmol m^−2^ s^−1^. Strategies to improve tolerance to light stress should be developed because an increased light supply would improve root formation. Hydrangea cuttings have only limited capacity to transport assimilated carbon to the stem base, the site of root regeneration, where water availability appears critical. The lower capacity for sucrose utilization and reduced hexose availability in the stem base of the weaker-rooting cultivar ‘Clarissa’ did not limit adventitious rooting per se but points to other predominant factors. The positive hexose response in the strong-rooting cultivar ‘Caipirinha’ indicates a risk of carbohydrate limitation and potential for improving hydrangea rooting through sugar application. Future studies will focus on the role of plant hormones.

## Figures and Tables

**Table 1:** Effects of cultivar (C) and dark storage (DS) at two different temperatures on hexose levels and hexose/sucrose ratios in the stem base of *H. macrophylla* cuttings determined at zero and three days post insertion (dpi). Results of 2-factor ANOVA and Tukey test (*n* = 6) and significantly different mean values. Experiment 1.

Factor	Hexose (µmol g^−1^ FM)		Hexose/Sucrose Ratio	
	0 dpi	3 dpi	0 dpi	3 dpi
C	ns	ns (*p* = 0.07)	ns	***
DS	**	ns	*	ns
C x DS	ns	ns	ns	ns
‘Caipirinha’	ns	24.30 a	ns	12.40 a
‘Clarissa’	ns	15.20 a	ns	5.09 b
Unstored	27.17 a	ns	7.96 a	ns
DS 20 °C	5.31 b	ns	1.39 b	ns
DS 4 °C	5.19 b	ns	4.74 ab	ns

*, **, *** indicate significant effects per dpi at *p* levels of 0.05, 0.01, 0.001, respectively; a, b indicate significantly different mean values at *p* level of 0.05; ns, not significant.

## Data Availability

The original contributions presented in this study are included in the article/Supplementary Materials. Further inquiries can be directed to the corresponding author.

## Supplementary Materials

The following supporting information can be downloaded at: https://www.mdpi.com/article/10.3390/plants15060968/s1, Table S1—Effects of cultivar (C) and dark storage (DS) at two different temperatures on leaf color and rooting parameters of *H. macrophylla* cuttings determined at 24 and 31 days post insertion (dpi). Experiment 1; Table S2—Effects of cultivar (C) and dark storage (DS) at two different temperatures on carbohydrate levels (µmol g^−1^ FM) in the basal leaf (LF) and stem base (SB) of *H. macrophylla* cuttings determined at 0 and 3 days post insertion (dpi). Experiment 1; Table S3—Effects of cultivar (C), dark storage (DS) and PPFD during cultivation of *H. macrophylla* cuttings on leaf and rooting parameters determined at 31 days post insertion. Experiment 2; Table S4—Effects of cultivar (C) and dark storage (DS) of *H. macrophylla* cuttings at two different temperatures on relative chlorophyll contents (SPAD values) of leaves at 0 dpi (a) and 3 dpi (b). Experiment 2; Table S5—Effects of cultivar, dark storage at two different temperatures and PPFD on carbohydrates in *H. macrophylla* cuttings. Experiment 2; Table S6—Correlation matrix: Basal leaf sugars—Leaf chlorophyll, discoloration. Experiment 2.

## Abbreviations

The following abbreviations are used in this manuscript:

ABA	Abscisic acid
AR	Adventitious root
C	Cultivar
DS	dark-stored
dpi	days post insertion (planting)
US	unstored
