# Supplementary material for: Roles of Cultivar, Light and Carbohydrates in Rooting of Cuttings of Hydrangea macrophylla
Source: Plants (Basel). 2026 Mar 20;15(6):968. doi: 10.3390/plants15060968 (PMC13030014; doi:10.3390/plants15060968)
Supplement: Supplementary file 1 [file plants-15-00968-s001.zip › Table_S1.pdf]

Table S1. Effects of cultivar (C) and dark storage (DS) at two different temperatures on leaf color and rooting parameters of *H. macrophylla* cuttings determined at 24 and 31 days post insertion (dpi). Results of 2-factor ANOVA and Tukey-test (n = 5, each n consisting of 6 cuttings) and significantly different mean values. Mean values ± SE per combination of C and DS treatment are illustrated in Fig. 2. Experiment 1.

Dpi	Factor	Leaf Discoloration Index	Percentage of rooted cuttings	Root number	Length per root (cm)	Total root length (cm)
24	C	ns	******	******	*	******
	DS	***	****	ns	*	*
	C x DS	*	**	**	ns	ns
24	'Caipirinha'	ns	94.00 a	22.91 a	0.71 a	16.50 a
	'Clarissa'	ns	54.89 b	10.23 b	0.60 b	6.55 b
24	Unstored	1.50 a	93.33 a	ns	0.72 a	13.75 a
	DS 20 °C	0.76 b	70.00 b	ns	0.67 ab	11.79 ab
	DS 4° C	0.89 b	60.00 b	ns	0.58 bc	9.03 bc
31	C	ns	*****	***	**	******
	DS	ns	**	***	ns	*
	C x DS	ns	**	*	ns	***
31	'Caipirinha'	ns	100.00 a	25.64 a	1.38 a	35.10 a
	'Clarissa'	ns	62.67 b	15.31 b	0.98 b	17.47 b
31	Unstored	ns	95.00 a	27.38 a	ns	31.54 a
	DS 20 °C	ns	84.00 ab	19.66 b	ns	27.04 ab
	DS 4 °C	ns	65.00 bc	14.37 b	ns	20.28 bc

*,**,***,****,*****, ****** indicate significant effects at the specific dpi at *p* levels of 0.05, 0.01, 0.001, 0.0001, 0.00001, 0.000001, respectively; a, b, c indicate significantly different mean values at *p* level of 0.05; ns, not significant.
