# Supplementary material for: Roles of Cultivar, Light and Carbohydrates in Rooting of Cuttings of Hydrangea macrophylla
Source: Plants (Basel). 2026 Mar 20;15(6):968. doi: 10.3390/plants15060968 (PMC13030014; doi:10.3390/plants15060968)
Supplement: Supplementary file 1 [file plants-15-00968-s001.zip › Table_S2.pdf]

Table S2. Effects of cultivar (C) and dark storage (DS) at two different temperatures on carbohydrate levels (µmol g^{-1} FM) in the basal leaf (LF) and stem base (SB) of *H. macrophylla* cuttings determined at 0 and 3 days post insertion (dpi). Results of 2-factor ANOVA and Tukey-test (n = 6) and significantly different mean values. No significant effects were found for sucrose in leaves. Mean values ± SE per combination of cultivar and dark storage treatment are illustrated in Figure 3. Experiment 1.

Dpi	Factor	Glucose LF	Fructose LF	Starch LF	Glucose SB	Fructose SB	Sucrose SB	Starch SB
0	C	*****	ns	ns	ns	ns	ns	ns
	DS	ns	***	*****	*	***	ns	***
	C x DS	*	ns	*	ns	ns	ns	ns
0	'Caipirinha'	3.04 a	ns	ns	ns	ns	ns	ns
	'Clarissa'	0.99 b	ns	ns	ns	ns	ns	ns
0	Unstored	ns	1.40 a	5.61 a	13.43 a	8.73 a	ns	0.98 a
	DS 20 °C	ns	0.62 b	0.45 b	3.88 b	1.43 b	ns	0.37 b
	DS 4° C	ns	0.50 b	0.34 b	3.60 b	1.59 b	ns	0.20 b
3	C	**	ns	ns	*	ns	*	ns
	DS	ns	*	ns	ns	ns	ns	**
	C x DS	ns	*	ns	ns	ns	ns	ns
3	'Caipirinha'	20.58 a	ns	ns	12.58 a	ns	2.28 b	ns
	'Clarissa'	11.11 b	ns	ns	6.85 b	ns	3.84 a	ns
3	Unstored	ns	8.28 b	ns	ns	ns	ns	0.90 b
	DS 20 °C	ns	13.95 a	ns	ns	ns	ns	1.06 b
	DS 4 °C	ns	12.72 ab	ns	ns	ns	ns	2.52 a

*,**,***,****,***** indicate significant effects at the specific dpi at *p* levels of 0.05, 0.01, 0.001, 0.0001, 0.00001, respectively; a, b indicate significantly different mean values at the *p* level of 0.05; ns, not significant.
