# Supplementary material for: Roles of Cultivar, Light and Carbohydrates in Rooting of Cuttings of Hydrangea macrophylla
Source: Plants (Basel). 2026 Mar 20;15(6):968. doi: 10.3390/plants15060968 (PMC13030014; doi:10.3390/plants15060968)
Supplement: Supplementary file 1 [file plants-15-00968-s001.zip › Table_S3.pdf]

Table S3. Effects of cultivar (C), dark storage (DS) and PPFD during cultivation of *H. macrophylla* cuttings on leaf and rooting parameters determined at 31 days post insertion. Results of 3-factor ANOVA and Tukey-test (n = 3, each n consisting of 10 cuttings). No significant effects on the rooting percentage and no significant interactions between the three factors were found. Experiment 2.

Factor	Leaf yellow index	Leaf red index	Root number	Length per root (cm)	Total root length (cm)	Root FM (g)	Root DM (mg)
C	ns	ns	***	**	***	****	****
DS	ns	***	**	****	***	ns	ns
PPFD	**	******	*	*	**	ns	ns
'Caipirinha'	ns	ns	33.7 a	1.17 a	39.9 a	0.65 a	54.3 a
'Clarissa'	ns	ns	25.3 b	0.99 b	27.7 b	0.27 b	23.5 b
Unstored	ns	0.22 a	34.1 a	1.24 a	42.98 a	ns	ns
DS 20 °C	ns	0.11 ab	27.6 b	1.15 a	33.85 ab	ns	ns
DS 4° C	ns	0.06 bc	26.8 b	0.85 b	24.58 bc	ns	ns
L100	1.39 a	0.23 a	32.1 a	1.16 a	38.4 a	ns	ns
L50	0.75 b	0.03 b	26.9 b	1.00 b	29.2 b	ns	ns

L100, 100 µmol $m^{-2}s^{-1}$; L50, 50 µmol $m^{-2}s^{-1}$; FM, fresh mass; DM, dry mass; *,**,***,****,*****, ****** indicate significant effects at *p* levels of 0.05, 0.01, 0.001, 0.0001, 0.00001, 0.000001, respectively; ns = non-significant; a, b, c indicate significantly different mean values at *p* level of 0.05.
