# Supplementary material for: Roles of Cultivar, Light and Carbohydrates in Rooting of Cuttings of Hydrangea macrophylla
Source: Plants (Basel). 2026 Mar 20;15(6):968. doi: 10.3390/plants15060968 (PMC13030014; doi:10.3390/plants15060968)
Supplement: Supplementary file 1 [file plants-15-00968-s001.zip › Table_S4.pdf]

Table S4 - Effects of cultivar (C) and dark storage (DS) of H. macrophylla cuttings at two different temperatures on relative chlorophyll contents (SPAD values) of leaves at 0 dpi (a) and 3 dpi (b). Experiment 2.

Table S4a. At time of planting (0 days post insertion). Results of 2-factor ANOVA and Tukey-test ($p < 0.05$, n = 238 - 240). Experiment 2.

**Cultivar** ****		**Dark storage** ******		**Cultivar x Dark storage** *	
Cai	58.39 b	US	63.07 a	Cai US	62.93 a
Cla	60.32 a	DS 20 °C	55.76 c	Cai DS 20 °C	54.11 d
-	-	DS 4 °C	59.24 b	Cai DS 4 °C	58.12 bc
-	-	-	-	Cla US	63.21 a
-	-	-	-	Cla DS 20 °C	57.41 c
-	-	-	-	Cla DS 4 °C	60.35 b

Cai, 'Caipirinha'; Cla, 'Clarissa'; US, unstored, ns, non-significant; *, ****, ****** indicate significant effect at p levels of 0.05, 0.0001, 0.000001, respectively; a, b, c indicate significantly different mean values.

Table S4b. At 30 days post insertion. Results of 3-factor ANOVA and Tukey-test ($p < 0.05$, n = 109 - 120). Experiment 2.

**Cultivar** ****		**Dark storage** ******		**PPFD** ******		**Cultivar x Dark storage** **		**Dark storage x PPFD** *	
Cai	50.64 b	US	49.54 b	L100	49.13 b	Cai US	46.62 d	US L100	45.17 c
Cla	53.70 a	DS 20 °C	51.13 b	L50	55.20 a	Cai DS 20 °C	49.89 cd	US L50	54.01 b
-	-	DS 4 °C	55.84 a	-	-	Cai DS 4 °C	55.54 ab	DS 20 °C L100	48.52 c
-	-	-	-	-	-	Cla US	52.53 bc	DS 20 °C L50	53.70 b
-	-	-	-	-	-	Cla DS 20 °C	52.41 bc	DS 4 °C L100	53.81 b
-	--	-	-	-	-	Cla DS 4 °C	56.15 a	DS 4 °C L50	57.92 a

Cai, 'Caipirinha'; Cla, 'Clarissa'; US, unstored; L100, 100 µmol $m^{-2}s^{-1}$; L50, 50 µmol $m^{-2}s^{-1}$; *, **, *****, ****** indicate significant effects at p levels of 0.05, 0.01, 0.0001, 0.000001, respectively; ns, non-significant; a, b, c indicate significantly different mean values at the p level of 0.05.
